# Arthroscopic Posterior Bankart Repair Using the Wilmington Portal to Facilitate Suture Anchor Implantation

**DOI:** 10.1016/j.eats.2023.03.009

**Published:** 2023-06-26

**Authors:** Matias Hoffman, Johannes Barth

**Affiliations:** Department of Orthopaedic Surgery, Clinique des Cèdres, Grenoble, France

## Abstract

Posterior shoulder instability (PSI) accounts for 2% to 10% of all shoulder instability, with recurrent posterior subluxation being the most common type. One of the most important risk factors is the presence of an anterior humeral notch (so-called reverse Hill-Sachs lesion), and the most common lesion in PSI is a posterior labral lesion. When conservative treatment fails, surgery is recommended to provide long-term stability, manage pain, and enable a return to previous activity levels. Most posterior labral tears are treated by an arthroscopic posterior Bankart procedure. Visualization of the posterior aspect of the glenohumeral joint is technically challenging in this procedure. The instrumental portal is also a matter of concern because there is no rotator interval posteriorly for cannula placement. The purpose of this article is to propose a technique using a secondary posterolateral Wilmington instrumental portal to perform easy and reproducible placement of the posterior suture anchor at a 45° angle to the glenoid rim. We recommend implementing this technique in patients with painful PSI or with a type B2 lesion according to the Moroder classification.

Posterior shoulder instability (PSI) is a rare condition, accounting for just 2% to 10% of all shoulder instability,^1^ and is most common in young men with a mean age of 24.5 years.^2^^,^^3^ The most common type of posterior instability is recurrent posterior subluxation.^4^ The main risk factors for recurrent instability are age (<40 years), a diagnosis of epilepsy, and the presence of an anterior humeral notch (reverse Hill-Sachs lesion) greater than 1.5 cm^3^ in volume.^5^

In a systematic review of 512 shoulders by Longo et al.,^2^ the most common injury was a posterior labral lesion (50%), with bony lesions in 210 shoulders (25%), including 80 humeral head depression fractures (9.4%) and 17 glenoid fractures (2%). PSI, especially in patients with atraumatic lesions, causes multiple nonspecific symptoms, including pain, subluxation, and functional impairment, which complicate the diagnosis and management.^1^

Conservative treatment can be effective, but recurrent posterior dislocation rates of up to 65% to 80% have been reported in some series.^4^ When conservative treatment fails, surgery is recommended to provide long-term stability, manage pain, and enable a return to previous activity levels. Excepting large glenoid fractures and severe glenoid bone loss, most posterior labral tears are effectively treated by an arthroscopic posterior Bankart procedure.^1^ We recommend implementing this technique in patients with painful PSI or with a Moroder type B2 lesion.^6^^,^^7^

The first technical difficulty encountered is creating a viewing portal to properly visualize the posterior aspect of the joint; the second difficulty is creating an instrumental portal. In contrast to an arthroscopic anterior Bankart procedure, there is no posterior rotator interval for cannula placement, and it is important to preserve the thin infraspinatus tendon fibers. The standard posterior viewing portal (soft-point portal) is too tangential to the joint line and is inadequate when managing the posterior labrum. The challenge in this type of procedure is to achieve an adequate arthroscopic approach angle for ideal anchor placement, as well as correct manipulation of the posterior glenoid rim, while maintaining a good view of the joint and minimizing iatrogenic cartilage damage. Morgan et al.^8^ showed that the anchor trajectory in SLAP lesions can be optimized with the addition of the posterolateral portal of Wilmington.^9^^,^^10^ We propose using this secondary instrumental portal so that a 45° approach angle to the posterior glenoid rim can be achieved (Video 1). Table 1 presents the steps, pearls, and pitfalls of this technique.

**Table 1:** Steps, Pearls, and Pitfalls of Bankart Repair of Posterior Labral Tears Using Posterolateral Wilmington Portal

Surgical Step	Tips and Pearls	Pitfalls
Creation of Wilmington portal	With the aid of a switching stick, change the viewing portal to the anterior superolateral portal. Use an 18-gauge spinal needle to find the optimal portal position. Enlarge the portal using a curved Halstead clamp.	Inappropriate portal positioning will not allow the ideal suture anchor angle of approach.
Labral preparation	Gently tap a curved 15° elevator (Arthrex) with a hammer when detaching the labrum.	The use of a radiofrequency electrocautery device will inhibit labral healing to the glenoid rim.
Anchor positioning and insertion	Introduce the curved 15° spear guide for FiberTak with the protection of the white plastic inserter to avoid soft tissues damages when penetrating the spear guide through the skin incision to the joint. Start by placing the first anchor in the most inferior part of the lesion. When drilling, perform a back-and-forth movement at least 3 times to ensure correct positioning. When inserting the anchor, do not push it from the tip; instead, insert it by holding the metal guide up to the drill hole in the bone. Note that the soft anchor is correctly inserted when the inserter handle is flush with the back of the spear guide.	The anchor metal guide is quite thin, and if tapped in from the anchor tip, it may break when entering the glenoid rim.
Labral repair	When the anchor is correctly placed, pull it to secure it. Use the black mark on the blue suture to pass it through the labrum and later to block it. Use the KingFisher retriever to reduce the labrum well before locking the anchor completely.	

## Surgical Technique

### Preoperative Evaluation and Indications

The patient undergoes an appropriate clinical examination and imaging of the glenohumeral joint, including radiography, magnetic resonance imaging, or computed tomography arthrography. Possible indications for performing posterior labral repair using the posterolateral Wilmington portal are isolated painful PSI or a type B2 lesion according to the Moroder classification (ie, painful lesion with functional impairment of structural dynamic posterior instability) without a reverse Hill-Sachs defect, posterior bony lesion, or pre-existing chondral damage.^6^^,^^7^

### Patient Positioning, Arthroscopic Portal Creation, and Initial Joint Exploration

**Fig 1:**
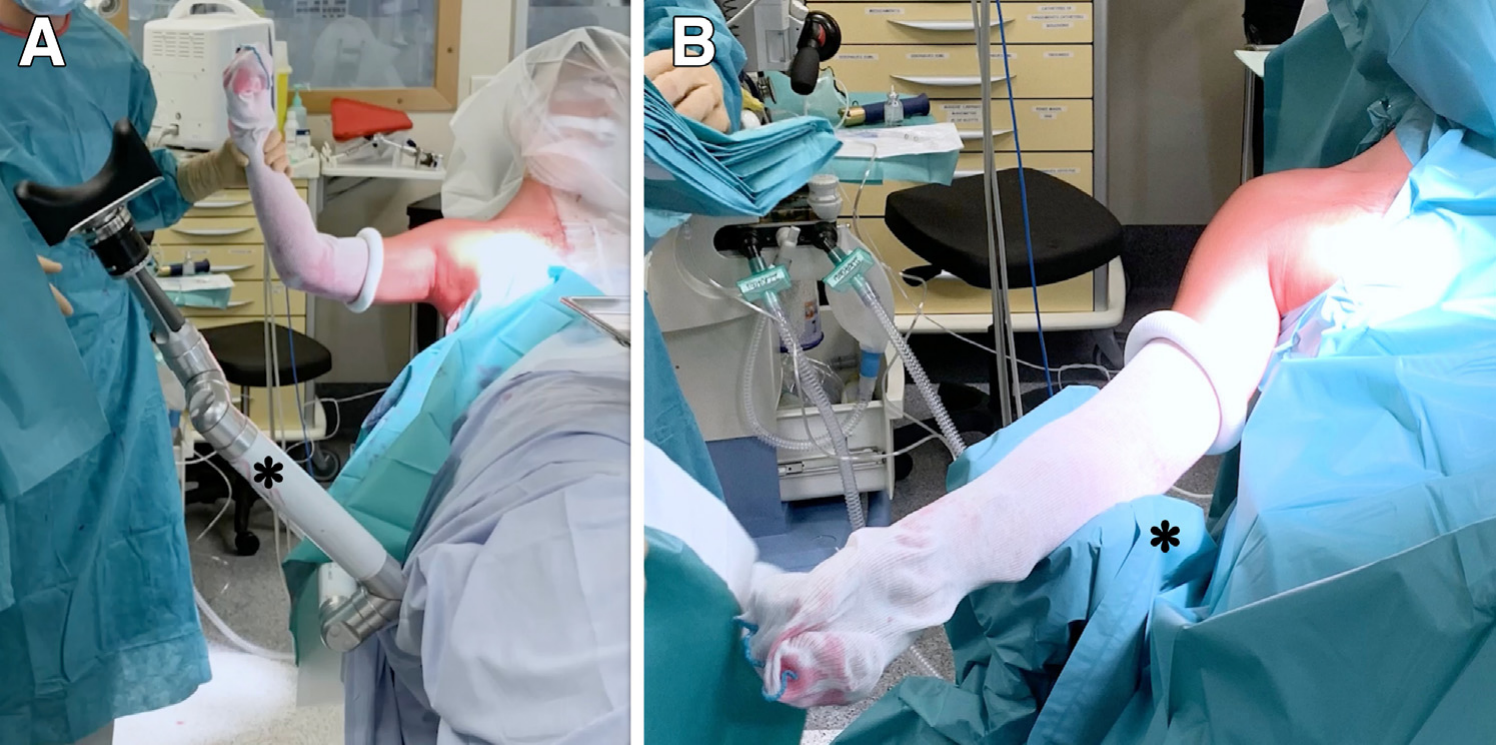
(A, B) Patient positioning for shoulder stabilization of right shoulder: beach-chair installation with arm placed freely on movable support (Trimano Fortis, asterisks).

The procedure is performed with the patient under general anesthesia in the beach-chair position with the arm placed freely on a moveable support (Trimano Fortis; Arthrex, Naples, FL) without traction. The patient’s head is secured in a head rest. At procedure commencement, the shoulder is placed in 30° of flexion and in neutral rotation (Fig 1).

**Fig 2:**
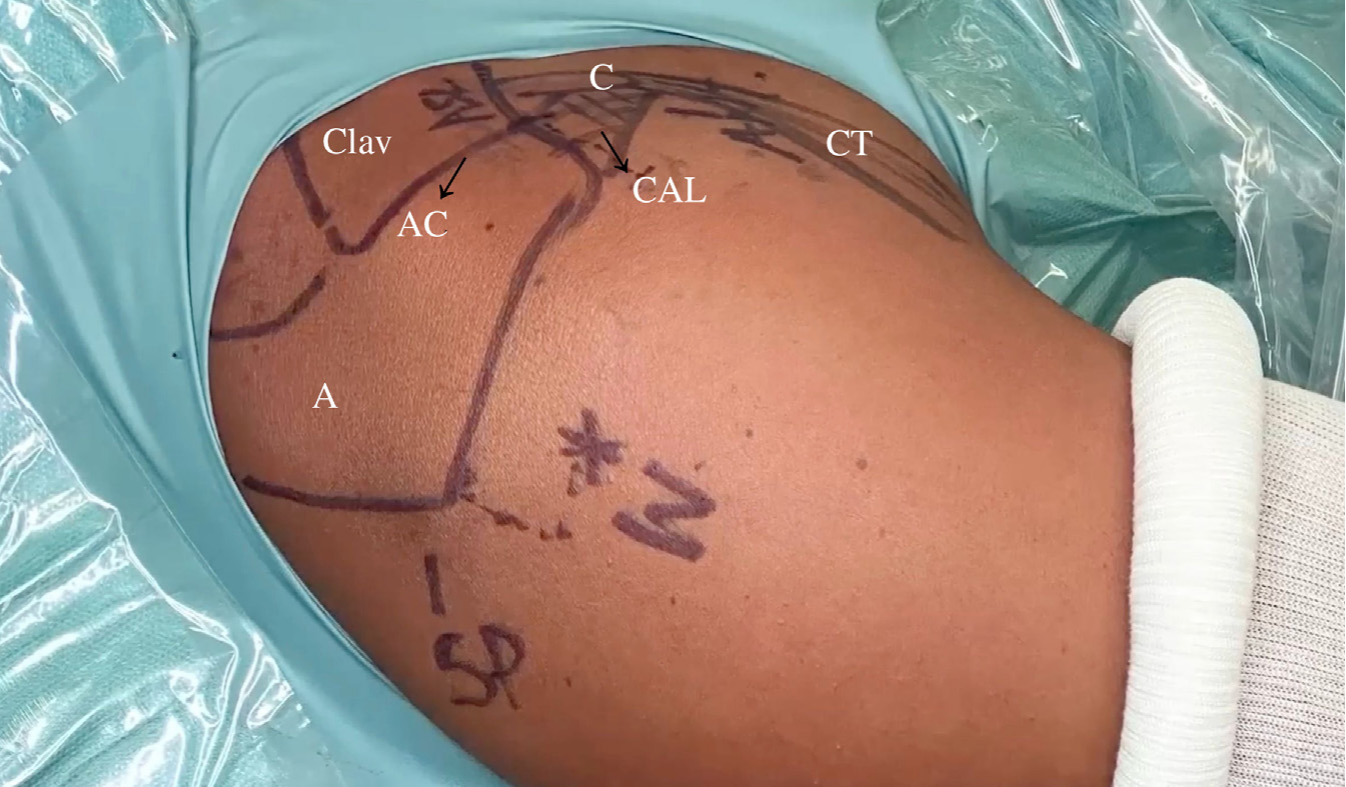
Bony landmarks and arthroscopic portals marked on right shoulder with patient in beach-chair position. (A, acromion; AC, acromioclavicular joint; ASL, anterior superolateral portal; C, coracoid process; CAL, coracoacromial ligament; Clav, clavicle; CT, conjoint tendon; SP, soft point; W, Wilmington portal.)

**Fig 3:**
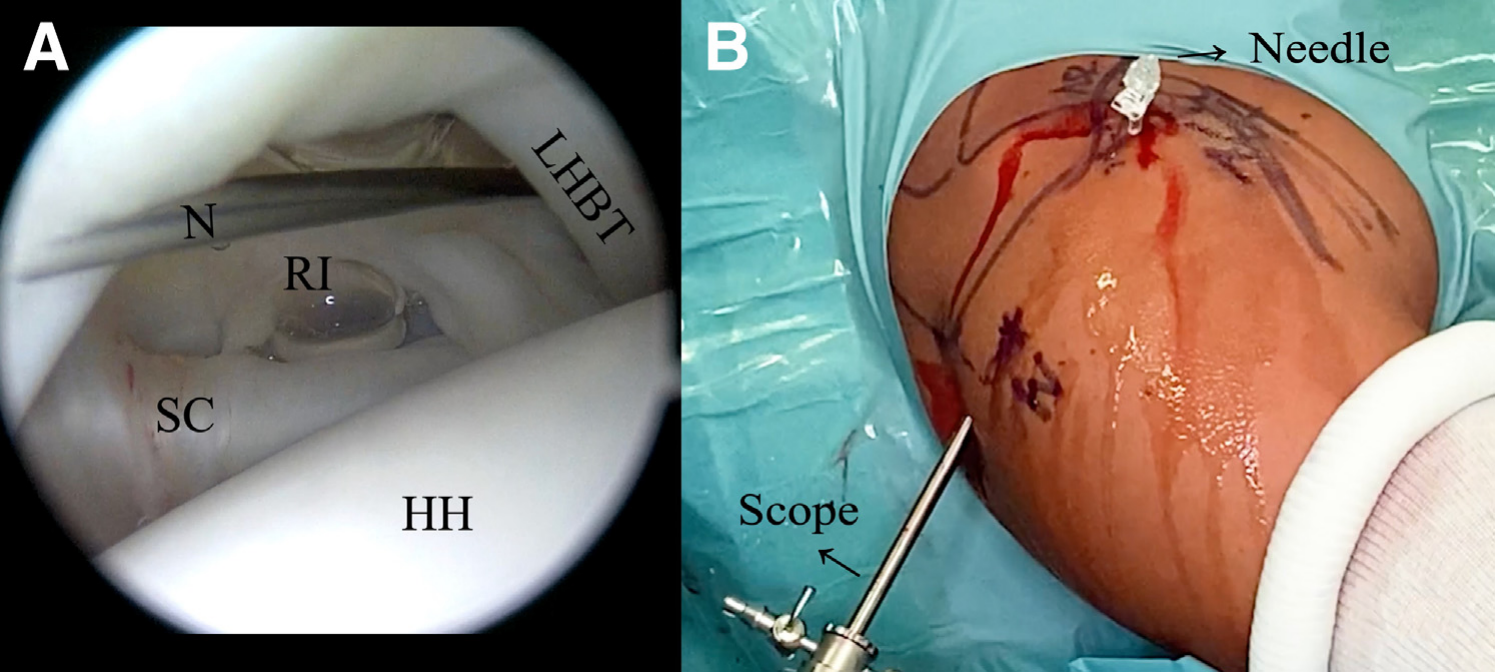
(A) Inside view from posterior (soft-point) portal in right shoulder with patient in beach-chair position showing anterior superolateral (ASL) portal creation using outside-in technique with 18-gauge needle (N). (B) Outside view with 18-gauge needle inserted into ASL portal and scope inserted into posterior (soft-point) portal in right shoulder with patient in beach-chair position. (HH, humeral head; LHBT, long head of biceps tendon; RI, rotator interval; SC, subscapularis tendon; W, Wilmington portal.)

**Fig 4:**
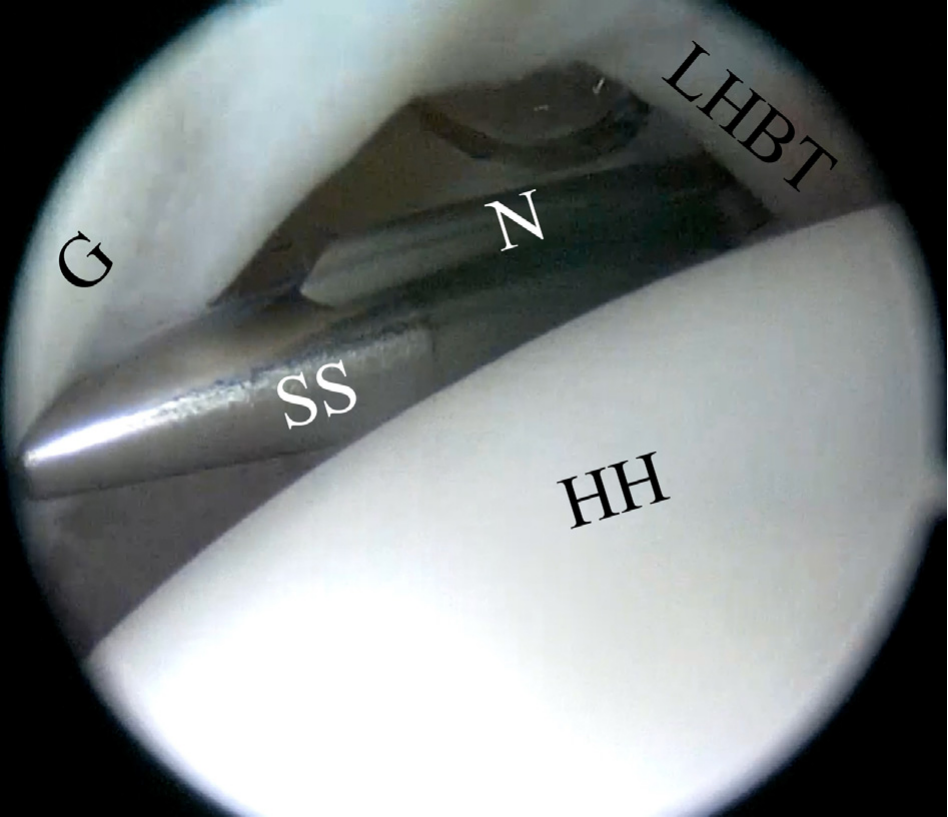
Posterior portal view, with switching stick (SS) and 18-gauge needle (N) inserted into anterior superolateral (ASL) portal to later—with help of switching stick—change viewing portal to ASL portal. (G, glenoid; HH, humeral head; LHBT, long head of biceps tendon.)

**Fig 5:**
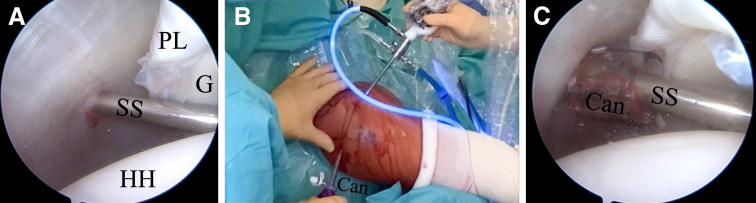
(A) Inside anterior portal view, with switching stick (SS) inserted into posterior (soft-point) portal to later introduce cannula through this portal. (B) Outside view of cannula (Can) insertion into posterior portal with help of switching stick. The arthroscope is inserted into the anterior portal. (C) Inside anterior portal view, with cannula (Can) already inserted into posterior portal and switching stick (SS) inside cannula. (G, glenoid; HH, humeral head; PL, posterior labrum.)

**Fig 6:**
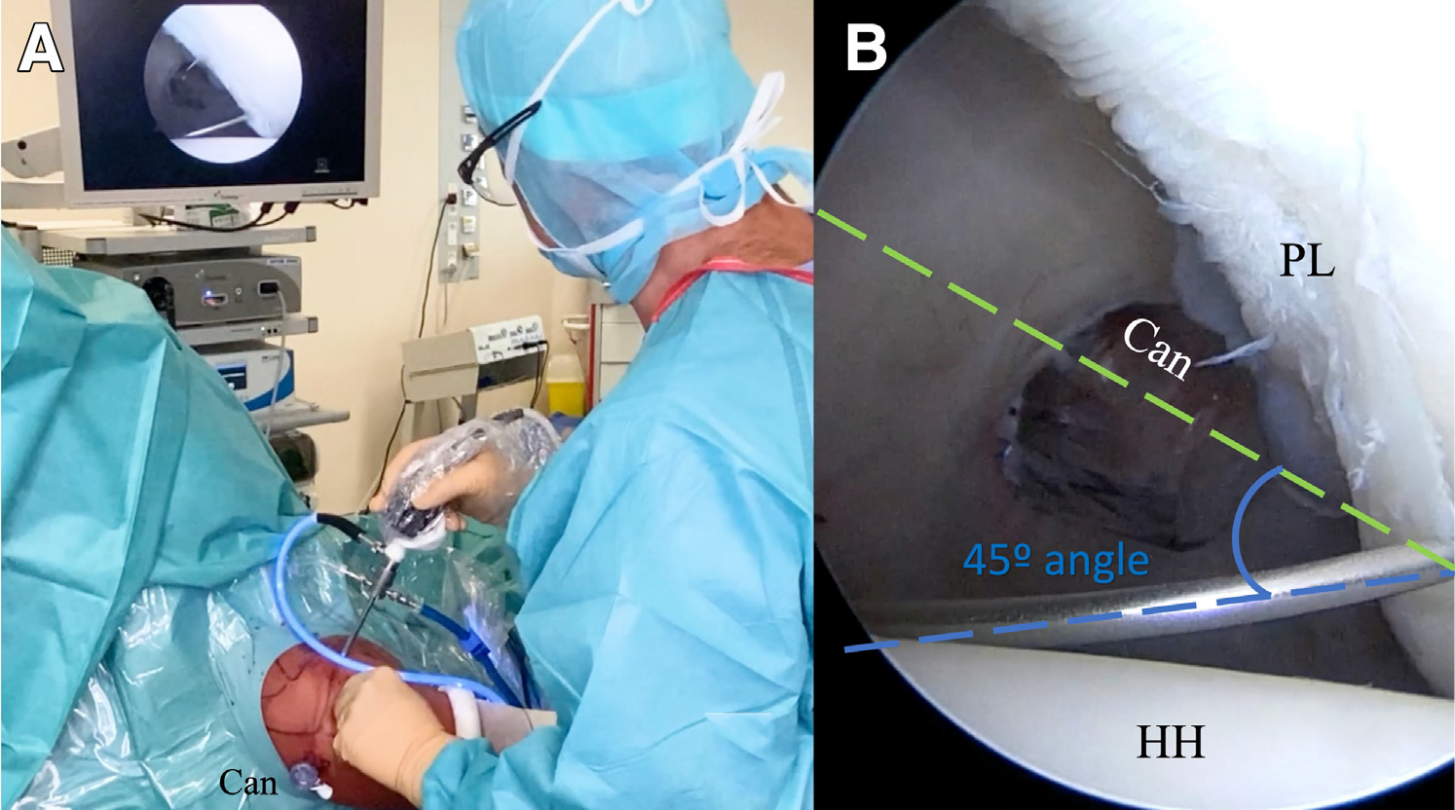
Right shoulder. (A) The arthroscope is placed in the anterior superolateral portal (ASL), and the cannula (Can) is inserted into the posterior (soft-point) portal. An 18-gauge spinal needle is introduced to create the Wilmington portal. (B) Achievement of 45° approach angle, with placement between posterior portal (green dashed line) and Wilmington portal (blue dashed line). (Can, cannula; HH, humeral head; PL, posterior labrum.)

The bony landmarks are drawn on the skin before the surgical procedure begins. The following 3 arthroscopic portals are necessary: posterior, anterior superolateral (ASL), and posterolateral (Wilmington), which is approximately 1 cm lateral and 1 cm anterior to the posterolateral border of the acromion (Fig 2). First, the standard posterior portal is created in the palpable soft point, and a 30° arthroscope is introduced into the glenohumeral joint. A thorough evaluation is performed, with confirmation of the posterior labral lesion, assessment of the glenohumeral cartilage, and assessment of the long head of the biceps tendon, as well as the superior labrum, anteroinferior labrum, and rotator cuff. The other 2 portals are created using an 18-gauge needle by an outside-in technique. The ASL portal is created just above the biceps tendon within the rotator interval and medial to the bicipital groove (Fig 3). Once the 2 aforementioned portals are made, we place a switching stick in the ASL portal (Fig 4) to switch the arthroscope to the ASL portal for the remainder of the procedure, which leaves the posterior portal to be used as an instrumental portal, in which we introduce a 7-mm rigid instrument cannula (Arthrex) under direct arthroscopic visualization (Fig 5). Finally, the posterolateral Wilmington portal is created using an 18-gauge needle positioned under vision 1 cm distal and 1 cm anterior to the posterolateral corner of the acromion, which allows a 45° approach angle to the posterior aspect of the glenoid (Fig 6).

### Labral Preparation

**Fig 7:**
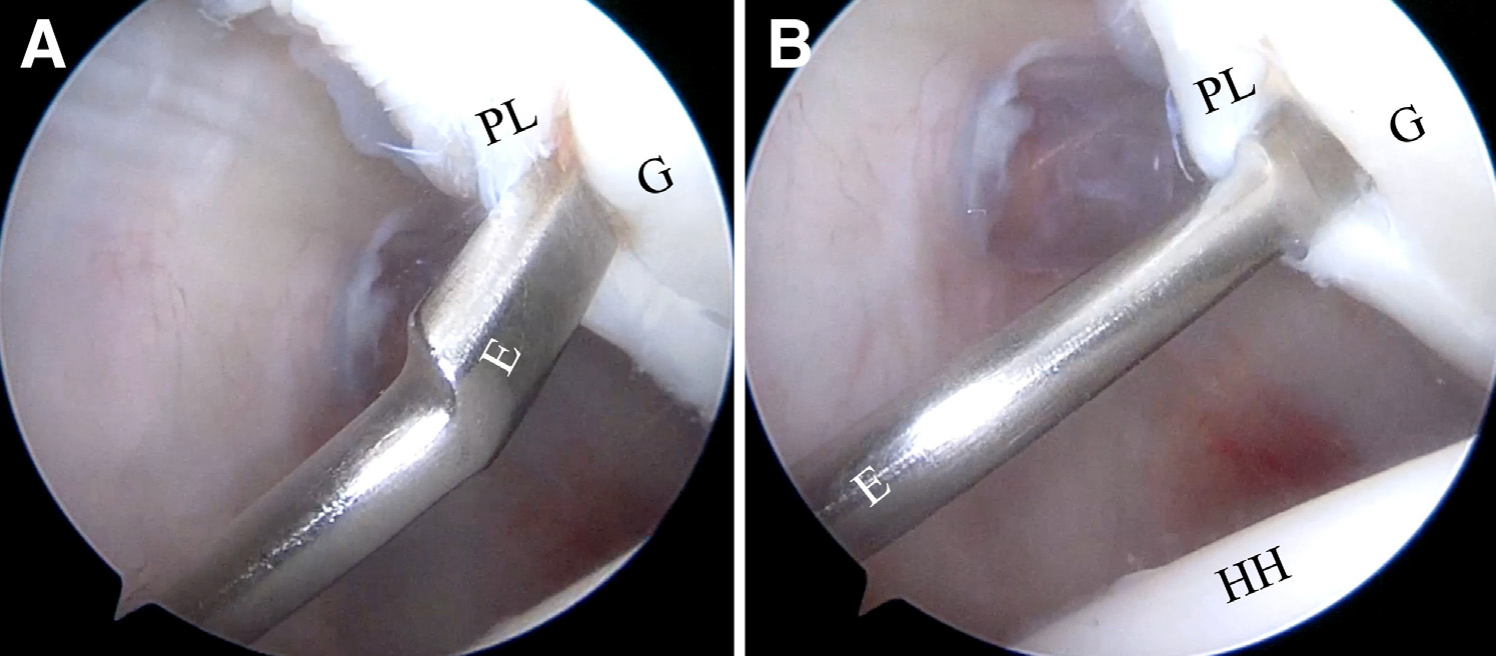
(A, B) Inside anterior portal view. Introduction of curved 15° elevator (E) through Wilmington portal to detach labrum from glenoid (G) rim. (HH, humeral head; PL, posterior labrum.)

**Fig 8:**
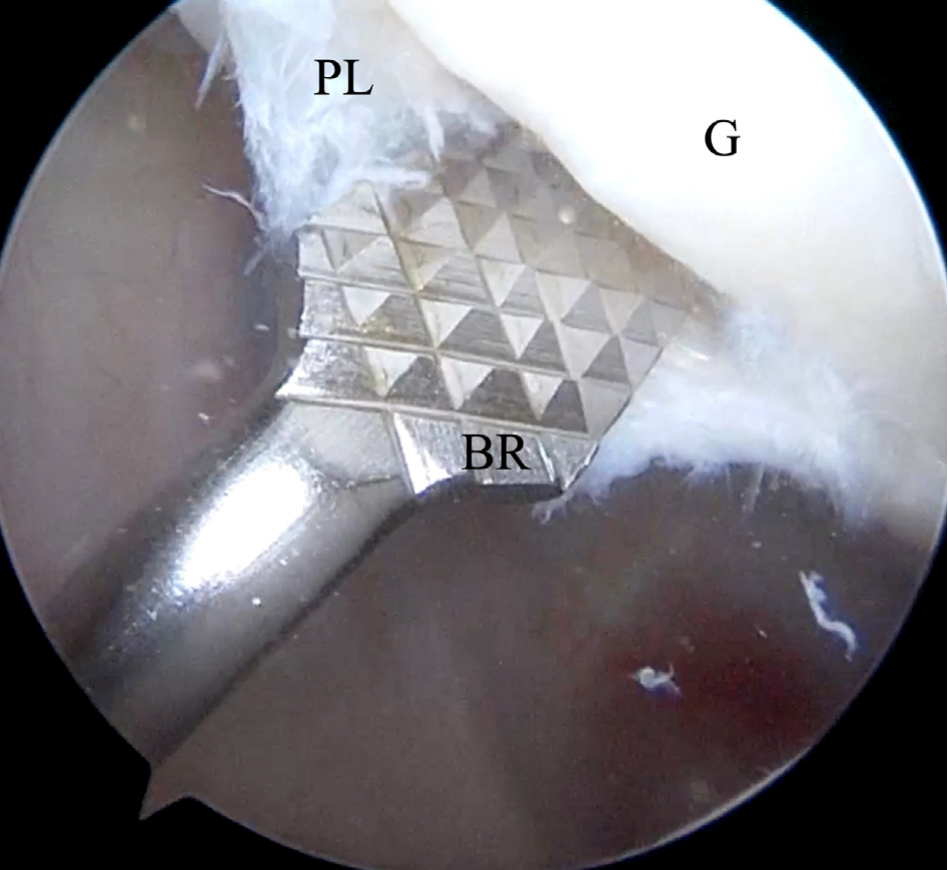
Inside anterior portal view. Insertion of bone rasp (BR) through Wilmington portal to abrade glenoid (G) side of capsulolabral complex. (PL, posterior labrum.)

After evaluation of the posterior labral lesion is completed, the arthroscope is placed in the ASL portal and a curved 15° elevator is introduced through the posterolateral Wilmington portal (Fig 7). Subsequently, a bone rasp is inserted and used as a tire lever on the capsulolabral complex and to abrade the glenoid rim (Fig 8). We recommend avoiding burning the soft tissue and bone with an electrocautery device because this may inhibit healing of the capsulolabral complex.

### Anchor Positioning and Insertion

**Fig 9:**
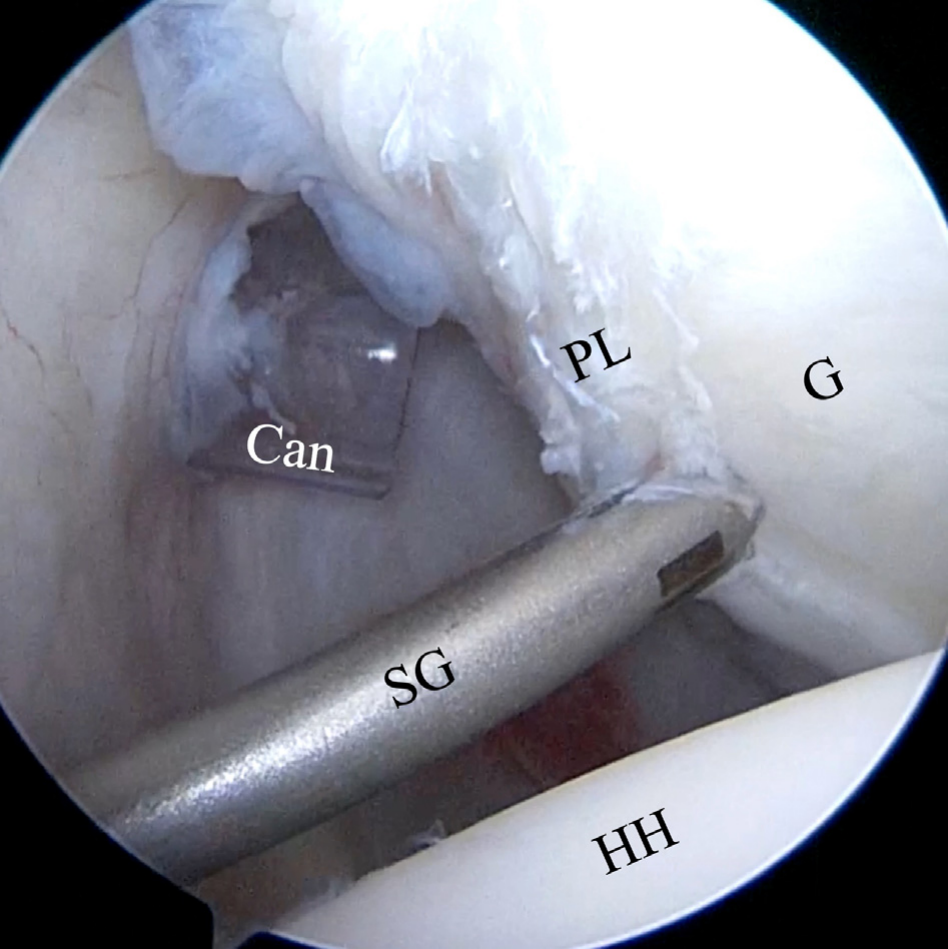
Inside anterior portal view. Positioning of curved 15° spear guide (SG) through Wilmington portal onto most inferior aspect of labral lesion at glenoid (G) rim. (Can, cannula; HH, humeral head; PL, posterior labrum.)

**Fig 10:**
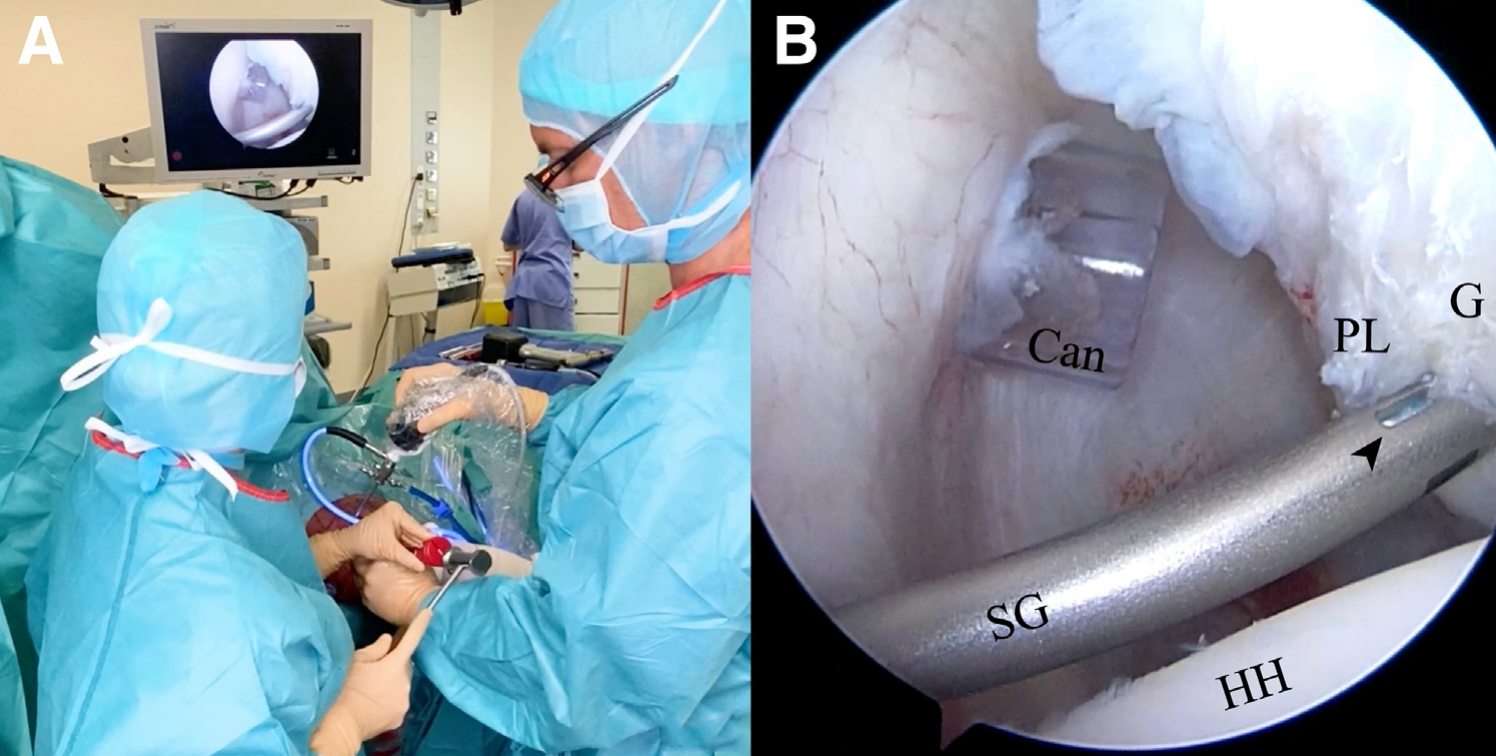
(A) Outside view of FiberTak suture anchor insertion. While the surgeon holds the arthroscope in the anterior portal with one hand, he uses the other hand to insert the curved 15° spear guide through the Wilmington portal, and the assistant introduces the suture anchor through the spear guide up to the glenoid (G) rim and completes anchor insertion with gentle impaction using a hammer. (B) Inside anterior portal view. The FiberTak suture anchor is inserted through the 15° spear guide (SG) at the most inferior part of the labral lesion onto the glenoid rim. The suture anchor insertion can be seen through the “window” of the spear guide (arrowhead). (Can, cannula; HH, humeral head; PL, posterior labrum.)

**Fig 11:**
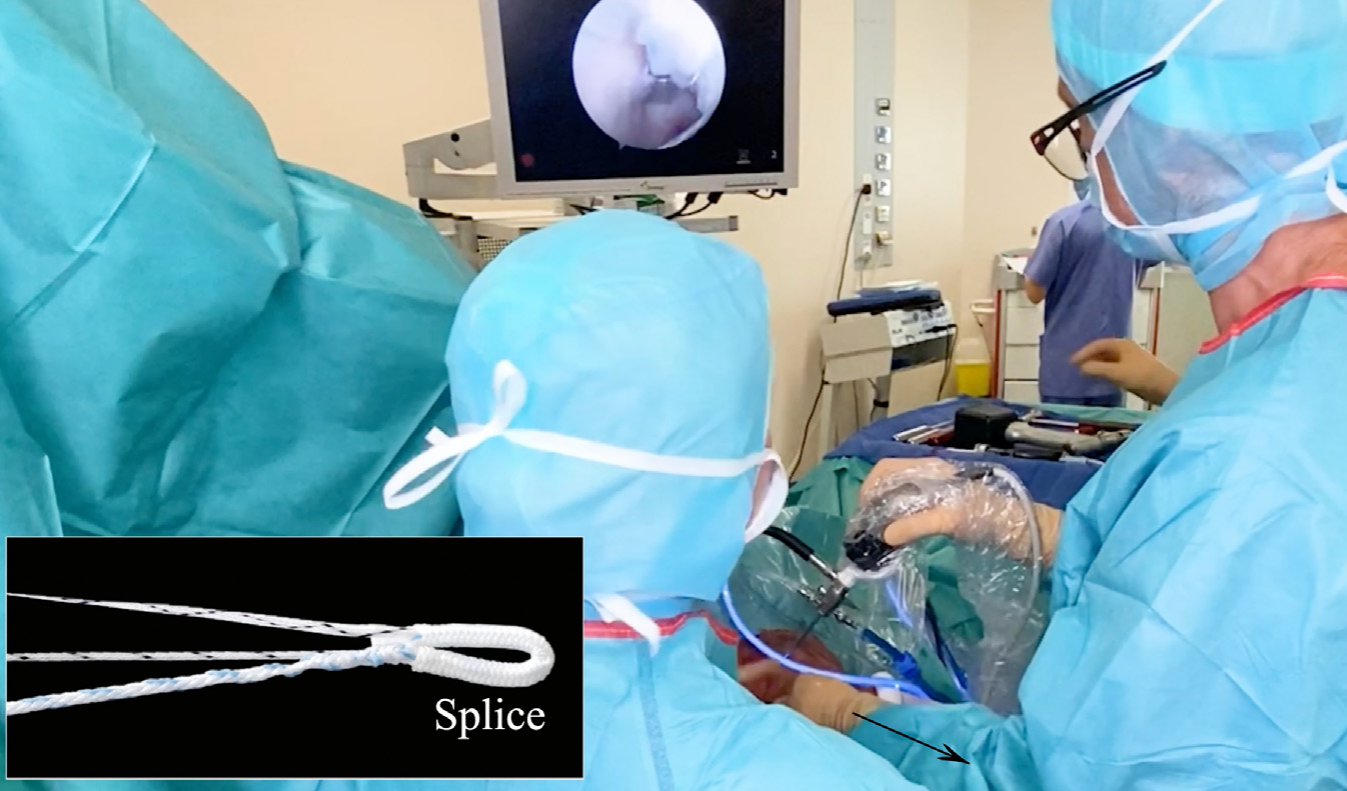
To confirm stable fixation of the anchor, the surgeon pulls on the sutures. This action will help first to confirm that the anchor is correctly inserted; finally, it will “adjust” the soft suture anchor’s splice (inset) in the glenoid rim to avoid pullout. The arrow indicates the direction of pullout.

We recommend using the smallest drill guide and soft knotless anchors possible, such as 1.8-mm FiberTak Soft Anchors (Arthrex). Through the Wilmington portal, a curved 15° spear guide is positioned onto the glenoid rim at the most inferior aspect of the lesion (Fig 9) to drill with a 1.8-mm flexible K-wire to the recommended depth marked by a laser line. The first FiberTak Soft Anchor is inserted by hand through the spear guide into the bone hole with gentle tapping (Fig 10). The suture-release tab is removed to release the sutures from the inserter, which is then removed along with the spear, and the sutures are pulled tight to confirm correct anchor placement (Fig 11).

### Labral Repair

**Fig 12:**
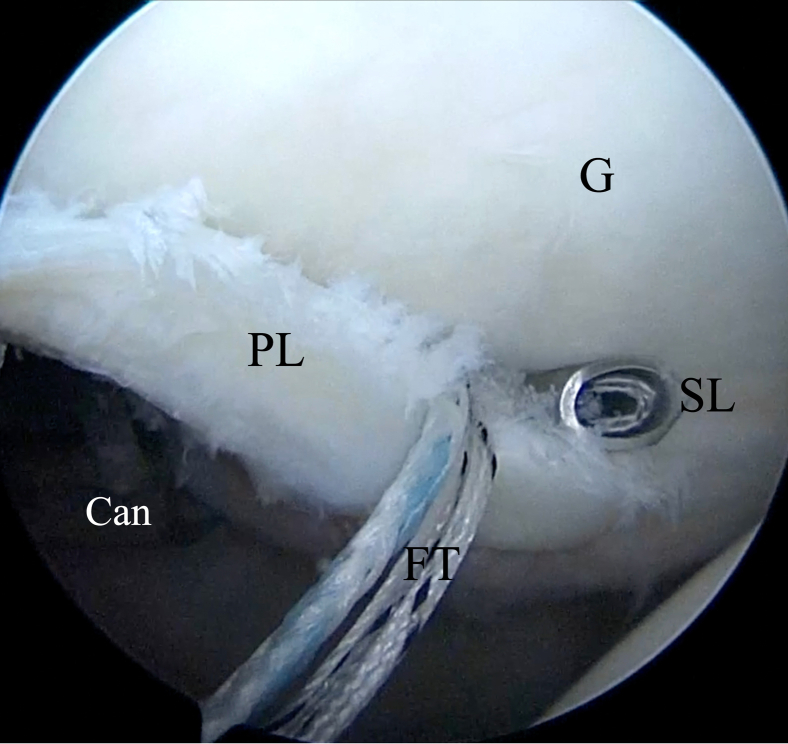
Inside anterior portal view. Insertion of SutureLasso suture passer (SL) through posterior portal (cannula [Can]) across capsulolabral tissue inferior to anchor to achieve capsular shift from inferior to superior and lateral to medial. (FT, FiberTak suture anchor; G, glenoid; PL, posterior labrum.)

**Fig 13:**
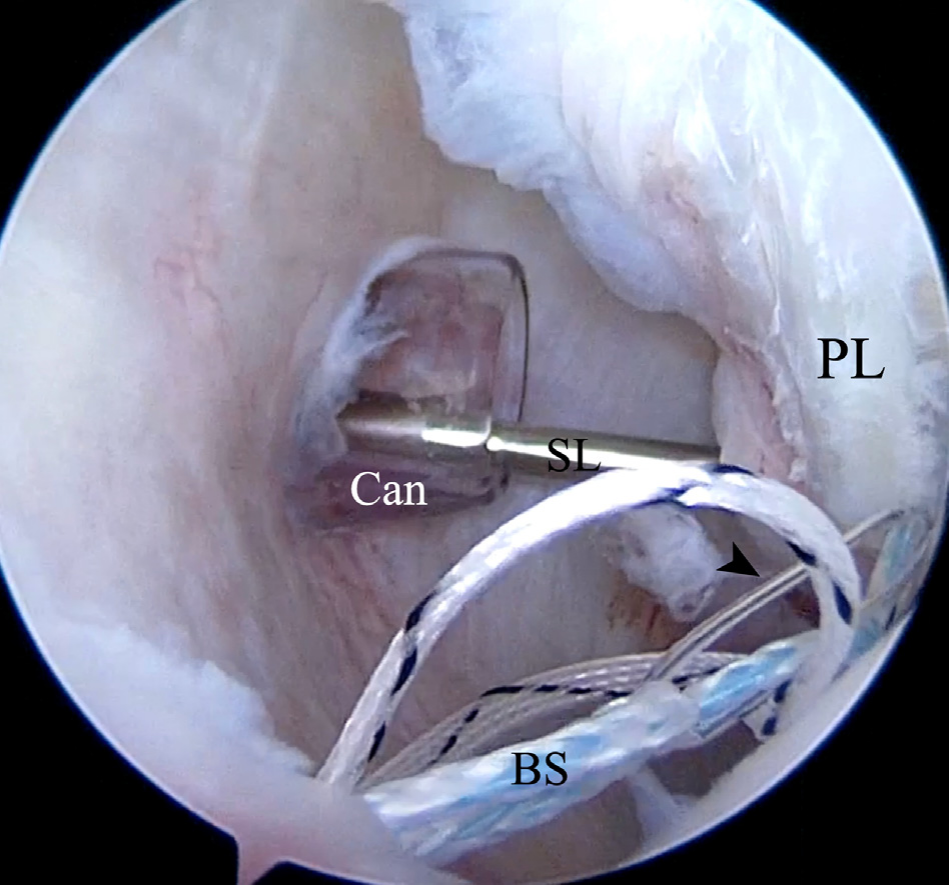
Inside anterior portal view. The nitinol wire loop (arrowhead) is advanced and the blue suture of the FiberTak suture anchor (BS) is inserted into the loop and pulled back to the Wilmington portal. Then, the blue suture is retrieved through the cannula. (Can, cannula; PL, posterior labrum; SL, SutureLasso.)

**Fig 14:**
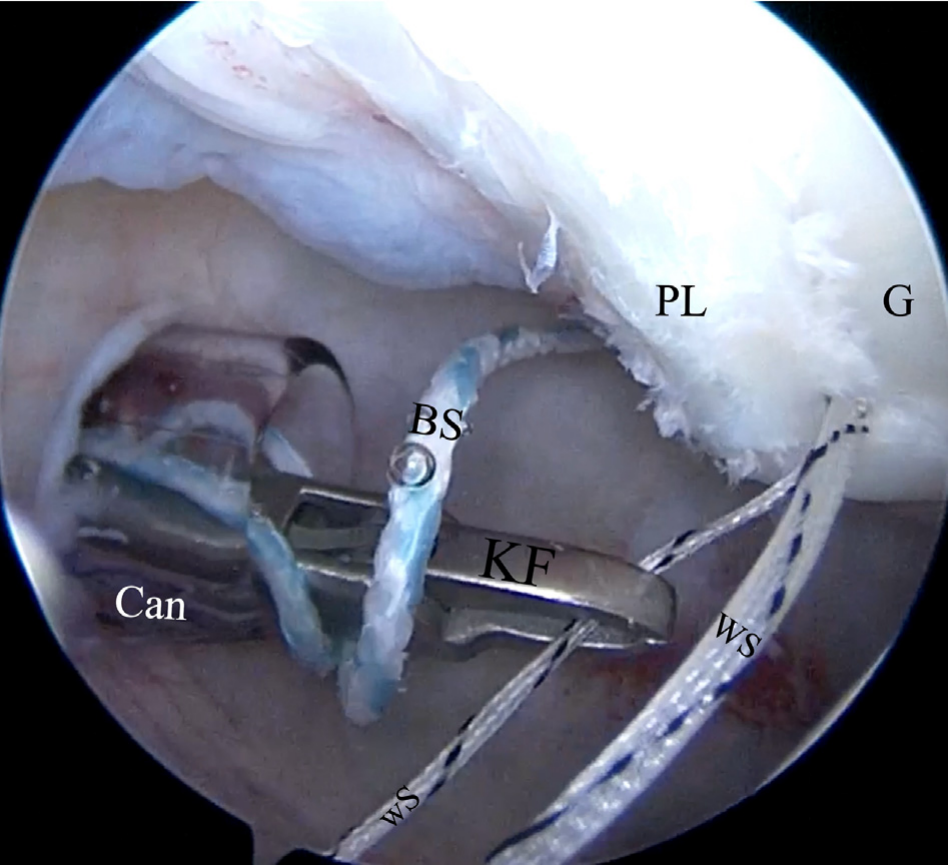
The KingFisher retriever (KF) catches the smaller white suture (wS), with the loop at its extremity of the FiberTak suture anchor (FT) back to the posterior portal. The tape is left in place. (cannula [Can]). (BS, blue suture of FiberTak suture anchor; G, glenoid; PL, posterior labrum; WS, white taped suture of FiberTak suture anchor.)

**Fig 15:**
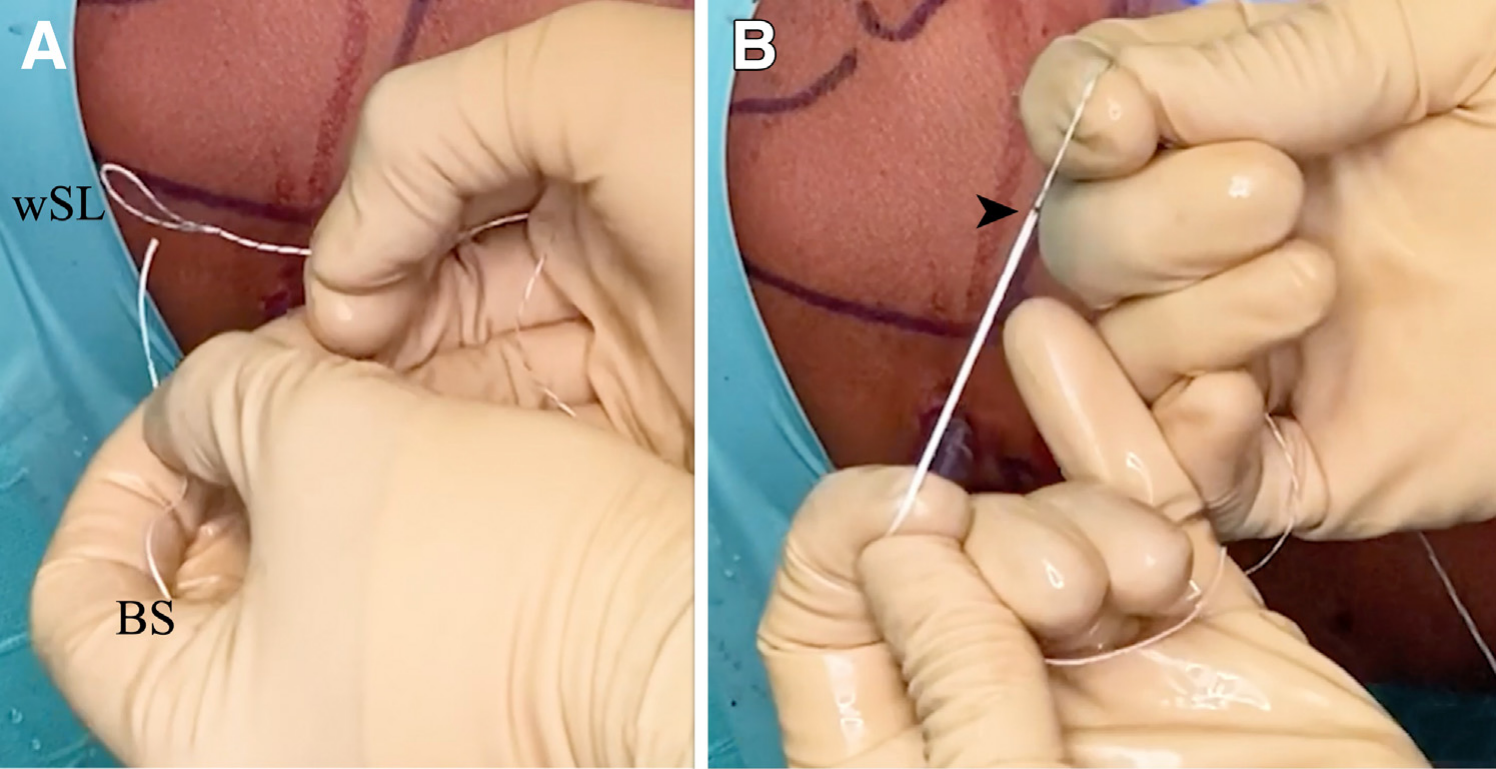
Outside view. (A) The blue suture (BS) is introduced into the small white suture loop (wSL). (B) The blue suture is introduced up to the black line (arrowhead) marked on the suture. This line indicates the correct length, which allows the surgeon to ensure that the suture is not going to be undone during passage of the blue suture across the labrum.

**Fig 16:**
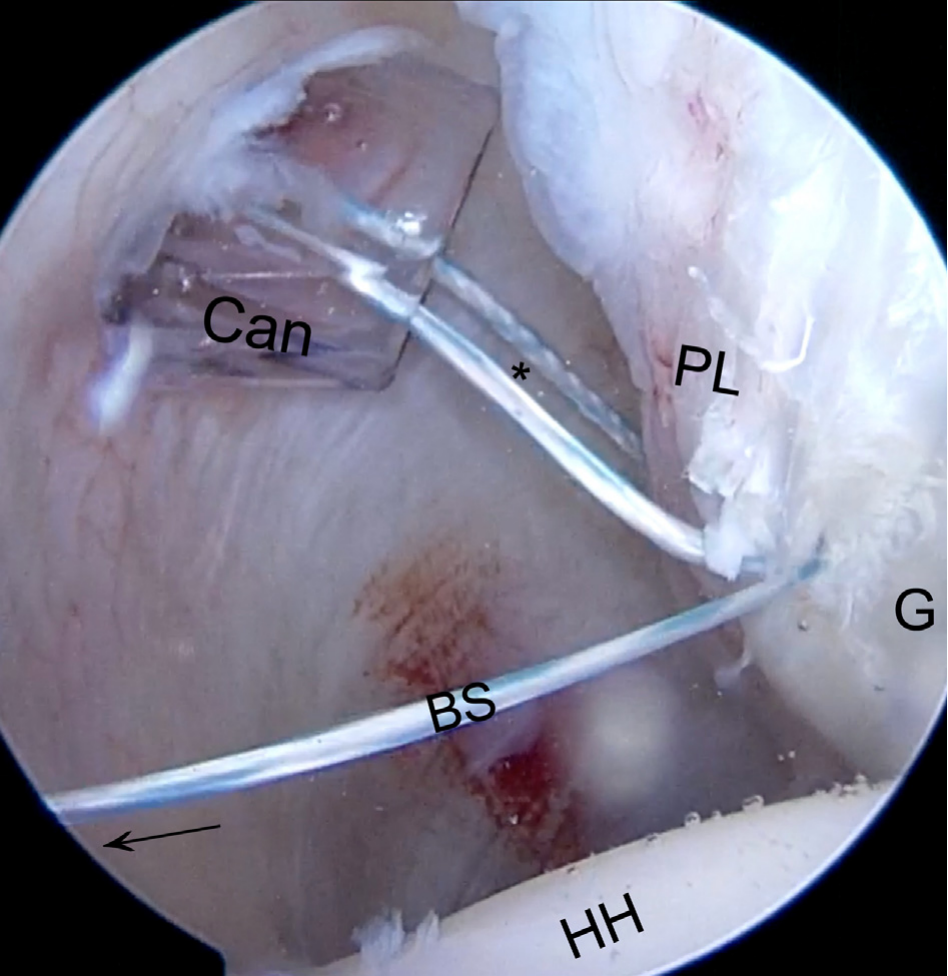
Inside anterior portal view. When the white taped suture is pulled out of the Wilmington portal (arrow), the blue suture (BS) is passed across the labrum and across the soft suture anchor’s splice. The loop (asterisk) that is formed around the labral complex can be seen. This loop is the one that will subsequently reduce the labrum onto the glenoid (G). (Can, cannula at posterior portal; HH, humeral head; PL, posterior labrum.)

**Fig 17:**
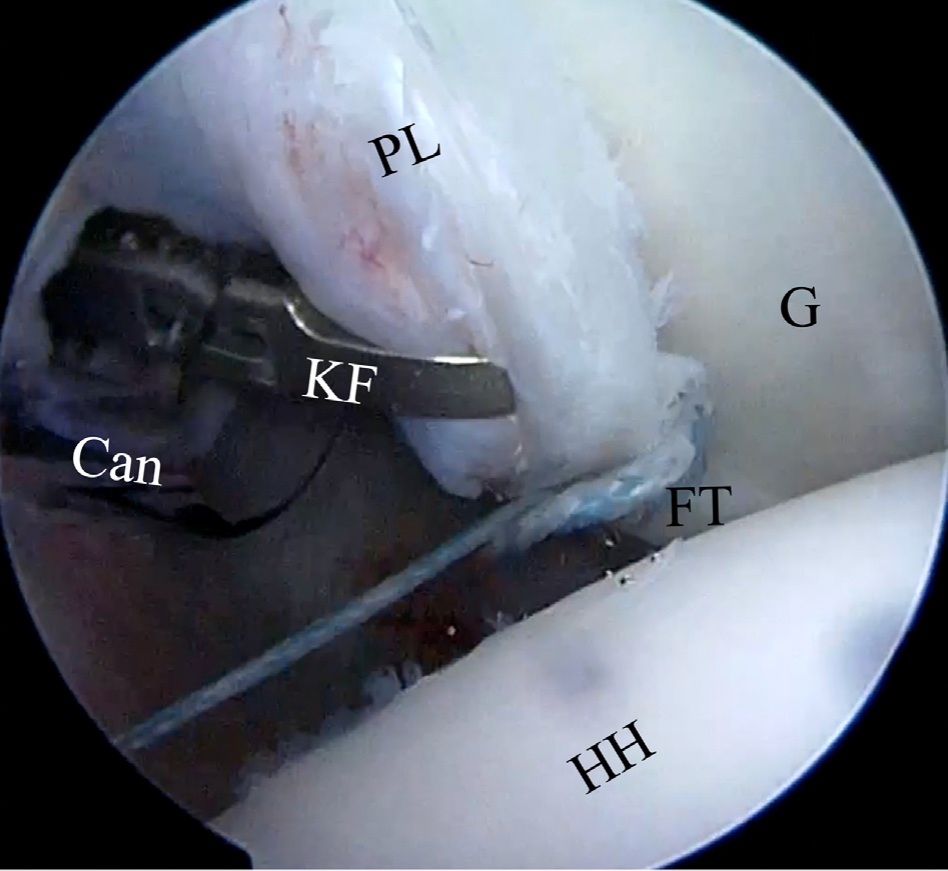
Inside anterior portal view. Before definitive tensioning of the soft suture anchor, it is important to grasp the labrum with the KingFisher retriever (KF) and reduce it into the desired position. (Can, cannula at posterior portal; G, glenoid; HH, humeral head; FT, final FiberTak suture anchor configuration; PL, posterior labrum.)

**Fig 18:**
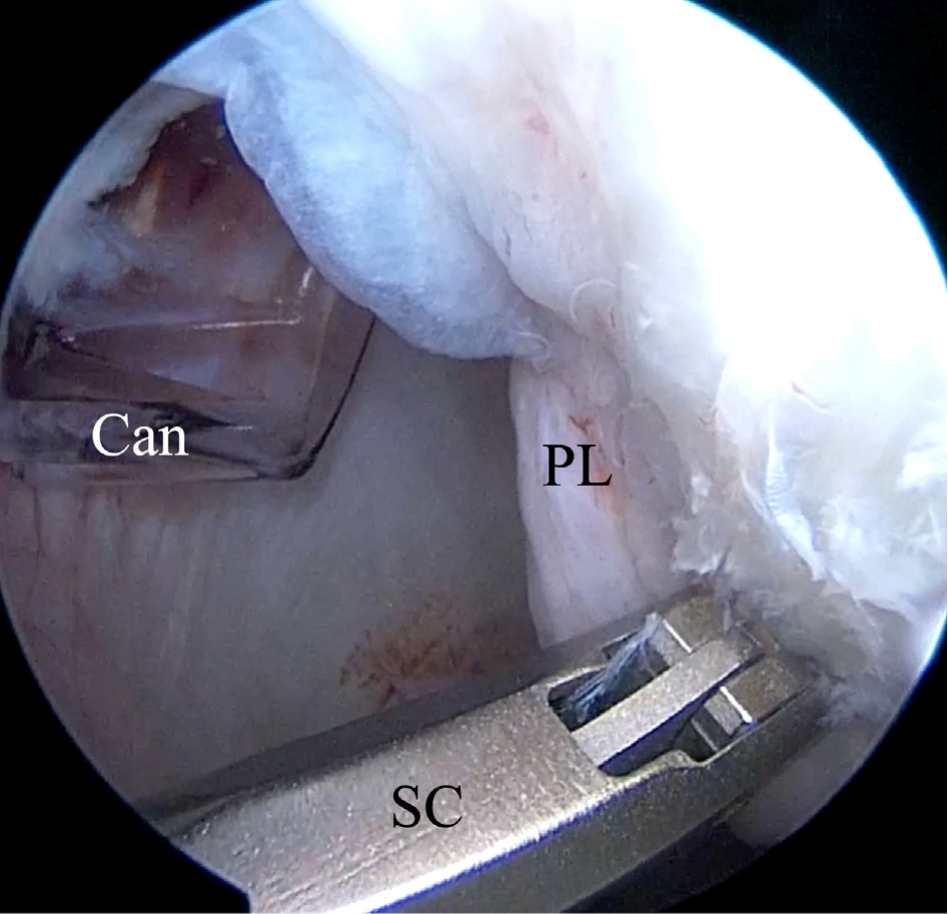
Inside anterior view. By use of a suture cutter (SC), the rest of the blue suture is cut flush to the labrum to obtain a low-profile repair. (Can, cannula; PL, posterior labrum.)

**Fig 19:**
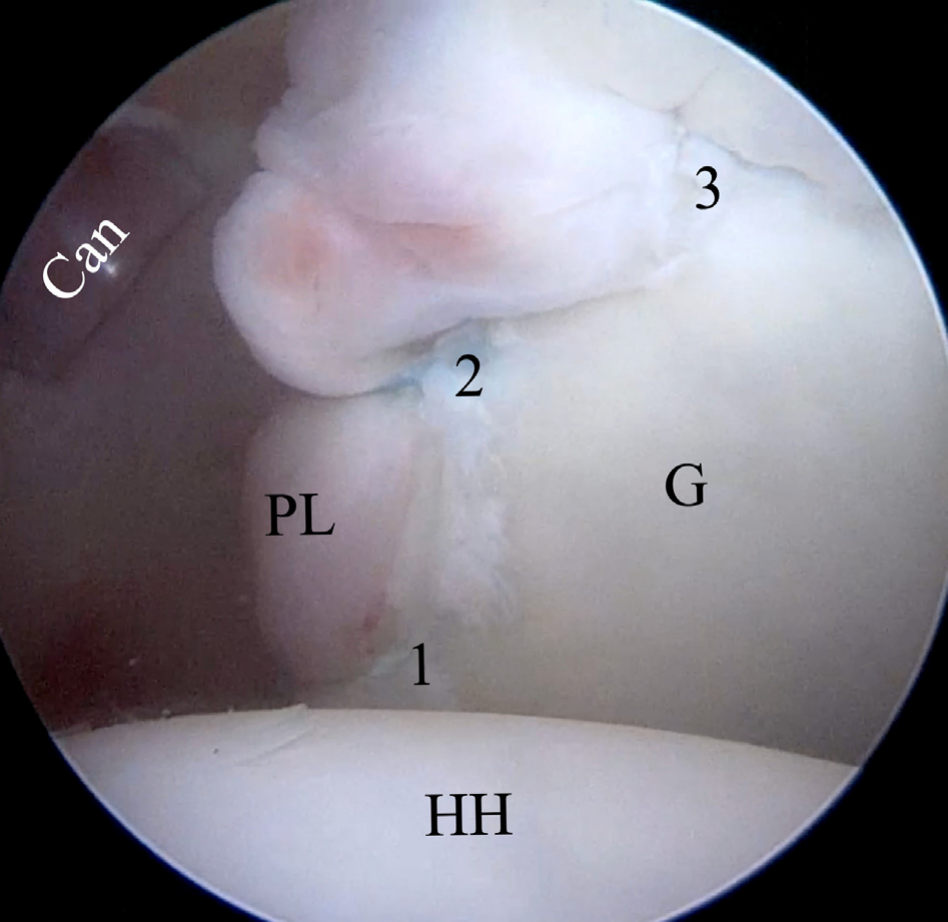
Inside anterior view showing final configuration in low-profile repair. In this case, we placed 3 suture anchors to obtain durable labral stabilization in the following order from inferior to superior: (1) lowest FiberTak Soft Anchor, (2) middle FiberTak Soft Anchor, and (3) highest FiberTak Soft Anchor. (Can, Cannula; G, glenoid; HH, humeral head; PL, posterior labrum.)

A Rotation Lasso Suture Passer (Arthrex) is introduced into the posterior portal to grab the capsulolabral tissue inferior to the anchor to achieve a capsular shift from inferior to superior and lateral to medial (Fig 12). The nitinol wire loop is advanced and the traction blue suture is passed through the loop (Fig 13) with a KingFisher retriever (Arthrex) introduced through the Wilmington portal. The blue suture loaded in the nitinol wire is pulled back to the posterior portal. The KingFisher retriever then catches the smaller white suture with the blue suture positioned through the posterior portal (Fig 14). Outside the shoulder, the blue suture is passed into the small white suture loop up to the black line marked on the suture (Fig 15), and the white suture tape is pulled from the Wilmington portal to pass through the labrum and the soft suture anchor (Fig 16). It is important that prior to identifying the appropriate amount of tension on the soft suture anchor, the surgeon should grasp the labrum with the KingFisher retriever and reduce it to the correct position (Fig 17). The surgeon may additionally use a knot pusher to adjust and secure the appropriate tension before cutting the excess suture flush to the labrum, obtaining a low-profile repair (Fig 18). This process is repeated as necessary to achieve stable fixation of the posterior labrum, and in our experience, 3 or 4 soft suture anchors are usually sufficient to achieve durable labral stabilization (Fig 19).

### Postoperative Rehabilitation

The patient is discharged from the hospital on the day of surgery with the arm in a sling for 4 weeks. A physiotherapy and hydrotherapy rehabilitation program is commenced at 3 weeks postoperatively following the Liotard protocol (immediate active auto-assisted rehabilitation) to recover shoulder range of motion. For the forward elevation, the patient is allowed to lift his arm actively (active auto-mobilization) but with the support of the controlateral arm (assisted) to avoid excessive load on the operated shoulder. Active external rotation is allowed, arm at the side, with the control of moderate pain (“Prech exercise”). Internal rotation is protected until 6 weeks, and then the recovery is left free. No lifting is allowed prior to 3 months postoperatively, and return to sport and heavy-duty activities is allowed after 4 months.

## Discussion

The primary purpose of this article is to propose the use of an accessory instrumental portal (Wilmington portal) to aid surgeons in achieving the ideal angle of approach for optimal suture anchor placement in the posterior glenoid, therefore reducing iatrogenic cartilage lesions or inadequate anchor placement. The angle of approach achieved through the soft-point posterior portal is almost parallel to the glenoid rim bone surface and therefore not ideal. Morgan et al.^8^ implemented the use of the Wilmington portal in the surgical management of type II SLAP lesions and achieved a 45° angle of approach for suture anchor placement to ensure bony purchase at the posterosuperior quadrant of the glenoid. In addition, in his theory of the deadman angle of suture anchors, Burkhart^11^ has already shown that the insertion angle should be less than 45° to increase anchor pullout strength and reduce suture tension, minimizing the chance of suture breakage. We decided to use the same portal to achieve optimal anchor placement and to achieve the ideal angle of insertion of the suture anchors on the posterior glenoid rim.

Our technique uses the ASL portal as a viewing portal, enabling the 2 posterior portals (soft point and Wilmington) to be used for instrumentation and suture management. With the use of this technique, the glenoid is viewed “front on” and triangulation is easier. Poehling-Monaghan et al.^12^ previously used an anterior rotator interval portal as a viewing portal for posterior Bankart lesion repair. With this method, the posterior shoulder is seen over the humeral head rather than “through” the glenohumeral joint.^12^ Moreover, Wolf and Eakin^13^ used an anterior viewing portal in their arthroscopic capsular plication technique to address PSI.

We prefer our approach in these patients because, as stated by DeLong et al.^3^ in 2015, it is a less invasive procedure, resulting in minimal disruption of the shoulder anatomy, improved visualization of intra-articular capsulolabral lesions, complete visualization of the intra-articular and subacromial spaces, and more precise, anatomy-specific repairs, as well as the ability to treat multiple articular lesions. In their systematic review, DeLong et al. also showed that arthroscopic techniques have superior outcomes to open techniques for unidirectional posterior instability of the shoulder regarding recurrence rate, subjective stability, patient satisfaction, return to sport at any level, and return to previous level of play. In a biomechanical study comparing arthroscopic posterior Bankart repair versus an open bone block procedure, Wellmann et al.^14^ showed that open repair with capsuloplasty tends to overcorrect posterior translation and does not effectively restore inferior stability. They recommended arthroscopic capsulolabral repair in cases of posteroinferior shoulder instability with verified capsulolabral lesions.^14^

Finally, we use knotless suture anchors with a flexible drill that facilitates better positioning and insertion of the suture anchors and always respect the 45° deadman angle to avoid suture anchor pullout. Such anchors are simpler and faster to adjust, and there is no difference between knotted and knotless suture anchors in the published literature. In a systematic review comparing knotted and knotless anchors for labral repair of the shoulder, Matache et al.^15^ concluded that on the basis of the available data, there is no significant difference in clinical outcomes between these 2 types of anchors. For Bankart repair, 3 studies found no biomechanical differences in load to failure or stiffness. Most of the studies in this review support the hypothesis that knotless anchors can restore labral morphology and function. This may be because of newer anchor designs that include curved drill guides, as well as the smaller profile of many knotless anchors, which facilitates anchor placement in the inferior portion of the glenoid rim. Another important finding from the aforementioned review is that knotless anchors can be used in Bankart repair in collision athletes, given that, according to Kocaoglu et al.,^16^ there is no difference in outcomes in collision athletes with knotless versus knotted anchors, with a 5% rate of recurrent instability in each group.^15^

Dey Hazra et al.^17^ described a technique using a single working portal for labral repair and capsular closure with knotless suture anchors with the patient in the beach-chair position, with the advantage of a simpler operative technique and reduced operative time. However, for less experienced surgeons, the use of a single portal to achieve the correct angle of approach to insert suture anchors may be challenging. Moreover, a second instrumental portal reduces the friction of suture passage during the use of a shuttle relay and during general suture management.

In conclusion, this article describes a reproducible technique for arthroscopic posterior Bankart repair using the anterior superolateral viewing portal to improve visualization of the posterior aspect of the labrum with 2 working portals. The soft-point posterior portal is used to perform suture shuttle relay and the Wilmington portal is used to facilitate suture anchor implantation with the optimal angle of approach.

## Supplementary Data

ICMJE author disclosure forms

Video 1 Surgical technique in patient aged 25 years with Moroder type B2 posterior labral lesion in right shoulder. The patient is placed in the beach-chair position. The condition of the rotator cuff, cartilage, long head of the biceps tendon, and cartilage is evaluated. The posterior labral lesion is then evaluated. The glenoid rim is debrided to stimulate healing of the capsulolabral complex to bone. Three 1.8-mm suture anchors are inserted into the posterior edge of the glenoid rim. The sutures are passed, and the labrum is repaired to the glenoid rim.
